# Dual Ionization Ion-Mobility Mass Spectrometry Hyphenated with Catalytic Oxygenation-Mediated Extraction

**DOI:** 10.1021/acsmeasuresciau.5c00160

**Published:** 2026-01-02

**Authors:** Tzu-Ching Tsai, Chamarthi Maheswar Raju, Pawel L. Urban

**Affiliations:** Department of Chemistry, 150417 National Tsing Hua University, 101 Section 2 Kuang-Fu Rd, Hsinchu 300044, Taiwan

**Keywords:** atmospheric pressure chemical ionization, green analytical chemistry, ion-mobility spectrometry, ionization methods, sample preparation, secondary electrospray ionization, volatile organic compounds

## Abstract

Catalytic oxygenation-mediated extraction (COME) is an environmentally friendly liquid–gas extraction technique that generates oxygen microbubbles via the catalytic decomposition of hydrogen peroxide. While corona discharge atmospheric pressure chemical ionization (APCI) is widely used for analyzing moderately polar and lower-polarity analytes with low molecular weights, secondary electrospray ionization (SESI) is a soft ionization technique that effectively ionizes polar volatile analytes. This study aims to integrate APCI and SESI with COME drift-tube ion-mobility (IM) triple quadrupole mass spectrometry (MS) to analyze volatile organic compounds (VOCs) with different physicochemical properties present in liquid matrices. The coupling of a house-built ion-mobility spectrometer with a commercial triple quadrupole mass spectrometer provides 2D separation at low cost. The user can choose one of the two ionization modes to achieve high signals with VOCs of different polarity. COME was applied to extract ethyl acetate from complex matrices (Taiwanese millet wine and whiskey) for immediate IM-MS analysis. An isotopically labeled internal standard was used to compensate for drift time and intensity shifts across multiple analyses. The system operates automatically with a graphical user interface enabling immediate ion-mobility spectrum visualization for targeted *m*/*z*.

## Graphical abstract

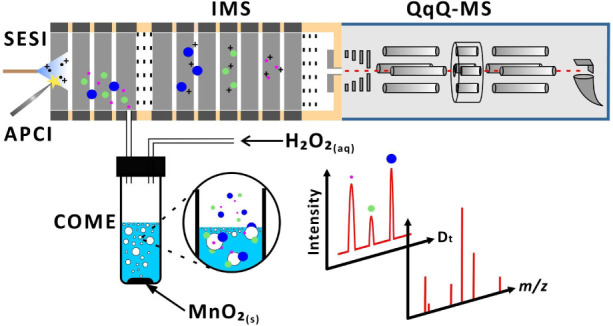

## Introduction

Ion-mobility spectrometry (IMS) is an analytical technique used to separate ionized molecules based on their size and shape.^1^ IMS is widely applied in diverse fields such as forensic science,^2^ food quality testing,^3,4^ environmental monitoring,^5^ and medical diagnostics,^6^ due to its selectivity and rapid analysis capabilities. By reporting the values of ion mobility (*K*) and reduced ion mobility (*K*_0_),^7^ IMS enables standardized measurements allowing for reliable comparisons across instruments and experimental conditions. *K* and *K*_0_ are given by the following equations:^8^

1 $$K=\frac{V_{d}}{E}=\frac{L}{t_{d}E}$$

where *V*_d_ is the drift velocity, *E* is the electric field, and *L* is the drift region length, and

2 $$K_{0}=K\frac{T_{0}}{T}\frac{P}{P_{0}}$$

where *T* is the drift-tube temperature, *P* is the drift-tube pressure, *T* is the temperature, *T*_0_ is standard temperature (273.15 K), and *P*_0_ is the standard pressure (760 Torr).

Over the years, multiple IMS techniques have been developed and applied to address the limitations of earlier designs.^9^ These developments have been driven by the need to improve resolution and selectivity, accelerate analysis speed, enhance compatibility with mass spectrometry (MS), and facilitate miniaturization and portability.^1,9,10^ Drift-tube IMS (DTIMS) is particularly popular due to its simplicity and acceptable resolving power, making it effective for separating and analyzing ions.^11^ Owing to its inherent working principle, IMS operates at atmospheric pressure, making it ideal for field applications such as detecting explosives and drugs.^12,13^ Advancements in miniaturization, portability, and cost-effectiveness have further enhanced its capabilities.^1,11^

Throughout the years, various ion sources have been employed in DTIMS and IM-MS to accommodate the diverse chemical properties and physical states of the analytes. These ionization methods include ^63^Ni or ^3^H radioactive ionization,^14,15^ atmospheric pressure chemical ionization (APCI),^4,16^ X-ray ionization,^17^ ultraviolet (UV) photoionization,^18^ and secondary electrospray ionization (SESI).^19–21^ For example, SESI ionizes volatile organic compounds (VOCs) by allowing neutral vapors to interact with highly charged primary electrospray droplets or newly formed reagent ions, transferring charge through proton transfer or adduct formation without direct sample contact.^22–26^ Moreover, in SESI-MS, polar VOCs generally exhibit higher ionization efficiencies (sensitivities) than less polar species.^23,27,28^ On the other hand, APCI is a soft ionization technique that ionizes the analytes through a corona discharge, generating reagent ions (such as protonated water clusters) that transfer the charge to the analytes via proton transfer or chemical ionization.^29^ APCI is ideal for analyzing the moderately polar and less polar low-molecular-weight compounds or VOCs.^30^ Dual ionization systems were also explored previously.^31^ As exemplified by the evaluation of low-temperature plasma, radioactive, and photoionization sources for IMS,^32^ it is important to compare different ionization sources within a given IMS configuration to assess their relative performance and suitability. Physically replacing ion sources within the experimental setup can be time-consuming and may introduce inconsistencies of the experimental conditions. Therefore, the availability of a fixed-configuration multimodal ion source is essential to improve the usability and analytical performance of IMS and IM-MS. Dual ion source systems offer advantages including reduced analysis time, improved experimental stability, enhanced analyte signal intensity, and a broader analyte detection range.^33^ The combination of SESI and APCI enables efficient analysis of both polar and nonpolar compounds. Considering these advantages, Jin et al. integrated a corona discharge and UV lamp dual ion source with a field asymmetric IMS system.^31^

Coupling IMS with MS provides enhanced analytical performance by furnishing complementary information. IMS separates ions based on their size, shape, and charge, while MS differentiates ions by their mass-to-charge ratio.^34^ When used together, ion-mobility (IM)-MS enables two-dimensional separation, greatly improving the resolution of complex mixtures and—in some cases—allowing for the differentiation of isobaric and isomeric species.^35,36^ This combined approach increases the confidence and specificity of compound identification, reduces chemical noise, and enhances signal clarity by separating coeluting or interfering species before mass analysis.^35,36^ IM separation cells are typically available only with high-resolution mass spectrometers, which are costly. For owners of less expensive low-resolution instruments, affordable house-built IM devices could provide a valuable way to enhance selectivity.^4^

Effervescence-based extraction methods have been shown to be useful green alternatives to conventional extraction systems.^37^ In catalytic oxygenation-mediated extraction (COME), the effervescence is generated by enzymatic catalysis, which rapidly decomposes hydrogen peroxide into water and oxygen, forming microbubbles, that facilitate the extraction of VOCs from the liquid matrix to the headspace.^38^ Here, we explore the integration of two ambient ion sources—APCI and SESI—with COME, an IM separation cell, and triple quadrupole (QqQ) MS (Figure 1, Movie S1). This dual ionization COME-IM-QqQ-MS system facilitates the detection of VOCs with diverse physicochemical characteristics through the use of both APCI and SESI ionization techniques. The usability of this system was demonstrated by analyzing selected real samples containing VOCs.

**Figure 1:**
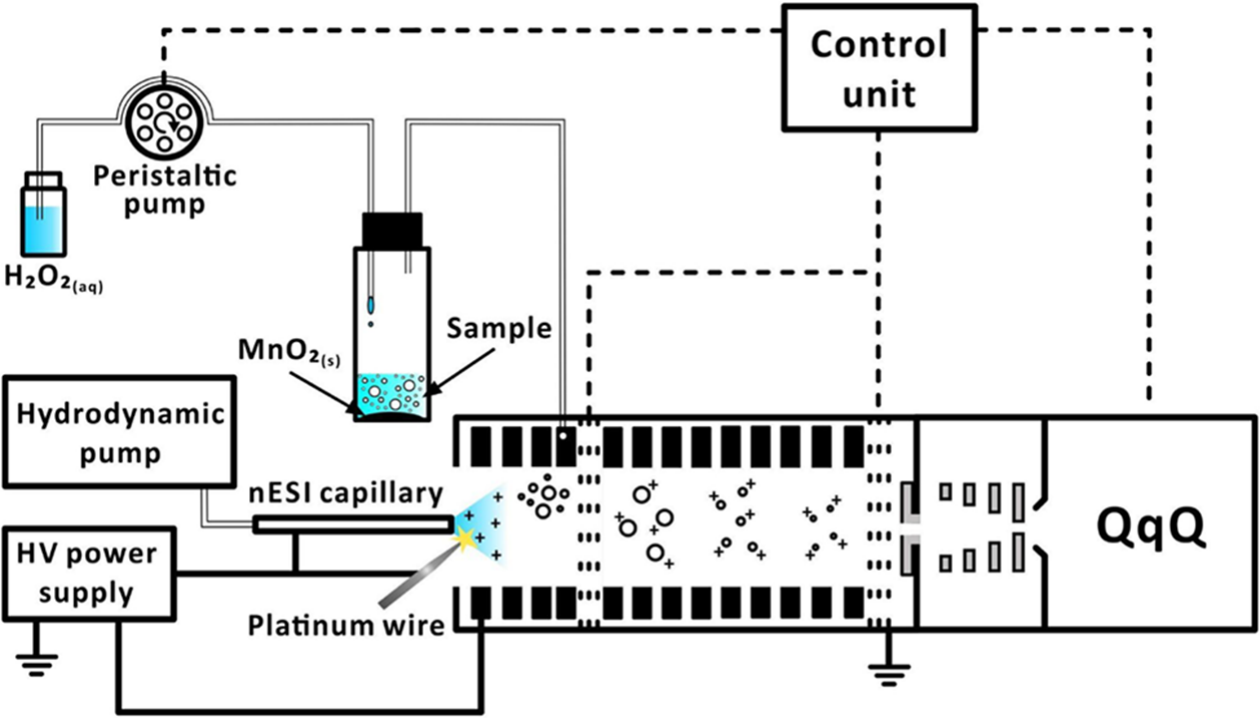
Schematic diagram of the dual ionization COME-IM-QqQ-MS system.

## Experimental Section

### Chemicals

Water (LC-MS grade) was purchased from Fisher Scientific (Hampton, NH, USA). Methanol (LC-MS grade) was purchased from Merck (Darmstadt, Germany). Ethanol (anhydrous, 99.5+%), hydrogen peroxide aqueous solution (35%, w/w), and (S)-(−)-nicotine (99%) were purchased from Echo Chemical (Miaoli, Taiwan). Ethyl acetate (>99.5%) was purchased from Avantor (Philadelphia, PA, USA). Acetic acid (ACS reagent, ≥99.8%) was purchased from Honeywell Fluka (Charlotte, NC, USA). Manganese­(IV) oxide (98%) was purchased from Alfa Aesar (Massachusetts, MA, USA). Traditional Taiwanese millet wine was purchased from a company brewing alcoholic drinks (Miaoli, Taiwan). Taiwanese whiskey (containing 40% ethanol) was purchased from a local liquor company (Taipei, Taiwan).

### COME System

The COME system consists of several integrated components. A peristaltic pump (ISM 404, MCP standard; Ismatec, Mount Vernon, IL, USA), fitted with Tygon tubing (length, 300 mm; ID, 1.59 mm; OD, 3.18 mm), was used for hydrogen peroxide solution delivery (Figure S1). One end of the Tygon tubing was connected to polytetrafluoroethylene (PTFE) tubing (length, 80 mm; ID, 0.8 mm; OD, 1.59 mm) to transfer hydrogen peroxide solution from a 50 mL glass sample bottle (Tung Kuang, Hsinchu, Taiwan). The opposite end was connected to PTFE tubing (length, 950 mm; ID, 0.3 mm; OD, 1.59 mm), followed by silicone tubing (length, 18 mm; ID, 1.0 mm; OD, 2.0 mm), and subsequently to the inlet tubing (length, 200 mm; ID, 0.5 mm; OD, 1.59 mm) leading to a 15 mL centrifuge tube (Thermo Fisher Scientific, Waltham, MA, USA) that served as the extraction chamber. The peristaltic pump flow rate was ∼264 μL min^–1^, and the total aqueous hydrogen peroxide solution (35%, w/w) volume of ∼572 μL was introduced over 130 s to produce oxygen microbubbles. The custom-designed extraction chamber cover was coupled with inlet and outlet PTFE tubings (length, 250 mm; ID, 0.8 mm; OD, 1.59 mm), which were connected to the peristaltic pump and the desolvation region of the IM separation cell (near the ion source), respectively. Prior to each analysis, the extraction chamber was loaded with 100 mg of manganese­(IV) oxide and 2 mL of LC-MS grade water. Subsequently, a 200-μL aliquot of the sample solution prepared in ethanol was introduced into the chamber. Oxygen microbubbles were then generated by pumping a hydrogen peroxide solution into the chamber for 130 s, where it was immediately decomposed by the manganese­(IV) oxide catalyst to facilitate extraction.

### Construction of a Drift-Tube Ion-Mobility Device

Inspired by the recent trend in prototyping instrumentation for chemistry,^39^ the drift-tube ion-mobility spectrometer was constructed using multiple components and electronic parts (Figure S2). The dual ion source holder was 3D-printed using acrylonitrile butadiene styrene material with a UP Plus 2 3D printer (Beijing Tiertime Technology, Beijing, China; Figure S3A, B). The APCI corona discharge needle was made of a 2.0 cm-long, 0.5 mm-thick platinum wire (stock no. 43288; Alfa Aesar, Haverhill, MA, USA) covered with a polyolefin insulation sleeve (Centenary Materials, Hsinchu, Taiwan), with only the needle tip exposed. The platinum wire was connected to a 20 kV power supply (MPS20P10/24/VCC; Spellman, Hauppauge, NY, USA). The nanospray electrospray ionization (nESI) emitter produced an ion spray plume for the SESI and was made of a fused silica capillary (length, 30 mm; ID, 0.02 mm; OD, 0.375 mm; GL Science, Tokyo, Japan), which was coupled with a metal union (UH-436; through hole, 0.15 mm; material, stainless steel; IDEX Health and Science), connected to another 20 kV power supply (MPS20P10/24/VCC; Spellman). The setup of the corona needle and nESI emitter is illustrated in Figure S3C. Stainless steel ring electrodes (Figure S4A and B), stainless steel ion gate electrodes (Figure S4C), and a stainless steel Faraday plate (Figure S4D) were fabricated by the NTHU workshop (Hsinchu, Taiwan). Note that, in this study, we have employed a different design of the drift-tube cell than in our previous study.^4^ It uses more durable materials (ceramics, steel electrodes) than the variant presented previously, which was based on PCB elements.^4^ In addition, the sample injection electrode was the last desolvation zone electrode (Figure S4B), and its 1.6 mm sample injection hole was offset 4 mm from the first ion gate electrode and connected to the extraction chamber through polytetrafluoroethylene tubing (Figure S1). The first electrode of the drift tube was powered by a 15 kV power supply (MPS15P10/24/VCC; Spellman). Ceramic spacers (Figure S4E) and ceramic drift-tube shells (Figure S5A, B) were fabricated by Shenzhen Hard Precision Ceramic (Shenzhen, China). A ceramic spacer was placed between each pair of ring electrodes to provide electrical insulation. It is important to note that, in each set of ion gate electrodes, electrodes nos. 1 and 3 were directly connected to the adjacent ring electrodes. A house-built hydrodynamic pump (Figure S6A)^40^ was used to pump electrolyte to the nESI emitter. Additionally, the relationship between the pump pressure and flow rate was evaluated using the optimized nESI electrolyte compositions (Figure S6B, C). The desolvation and drift region electrodes were connected in series with 0.5 MΩ and 1 MΩ resistors (Centenary Materials), respectively. The first ion gate electronic unit was constructed with 0.25 MΩ resistors in series (Centenary Materials), an MTS-1 3P3 mini toggle switch (Kinsten, Hsinchu, Taiwan), and a BNC female 90-degree adapter (Kinsten). A field-effect transistor (FET) pulser^41^ (GAA Custom Electronics, Kennewick, WA, USA) was used to control the pulse of the first ion gate. Moisture was removed from the nitrogen gas stream supplying the drift tube using a 5A molecular sieve tube (catalog no. 20618; Supelco, Sigma-Aldrich). A mass flow controller (model no. F-201CL-013–5K0-A; Bronkhorst, Ruurlo, Netherlands) was used to control the flow rate of drift gas (Figure S7A–D).

### Coupling of the Drift-Tube Ion-Mobility Device with a Triple Quadrupole Mass Spectrometer

The drift tube was coupled to the QqQ-MS (LCMS-8030, Shimadzu, Kyoto, Japan) via a custom-built ion transfer unit (Figure S8A), fixed with 2 polyetheretherketone components (Figure S8B, C), which were coupled with the MS inlet to enable the entry of ion packets into the vacuum compartment via the MS inlet. Additionally, the second ion gate was provisioned to control the transfer of the ion packets. The electronic circuit design of the second ion gate was constructed by 0.2 MΩ resistors in series (Centenary Materials), an MTS-1 3P3 mini toggle switch, and a BNC female 90° adapter (Kinsten). Please note that—in the second ion gate—the last grid electrode was connected to the ground and functions as the aperture grid. Another FET pulser (GAA Custom Electronics) was used to control the pulse of the second ion gate. A multifunctional control device (Analog Discovery 2 module; Digilent, Pullman, WA, USA) was used to set pulse parameters using JavaScript (pulse shape, frequency, amplitude, offset, symmetry, and phase). The ion gate pulse was incrementally shifted across a scanning range of 0.222 ms in the ion mobility spectrum every 2 s. For example, a complete scan from 0 to 20 ms required a total acquisition time of 180 s. A graphical user interface (GUI; Figure S9) was deployed on the Jetson Nano 2GB developer kit (NVIDIA Corporation, Santa Clara, CA, USA; Figure S7E,H) to control the maximum phase shift of gate opening (0° < end phase shift ≤ 360° corresponding to 0 ms < target analyte drift time ≤ 40 ms; waveform frequency, 25 Hz). The selected ion monitoring (SIM) mode QqQ-MS operating parameters are listed in Table S1.

## Results and Discussion

### Optimization of Dual Ionization COME-IM­(-QqQ-MS) Setup

For the purpose of optimization, a 200 μL aliquot of 1.5 × 10^–2^ M ethyl acetate in ethanol was pipetted into the extraction chamber containing 2 mL of water and 100 mg of manganese­(IV) oxide as the catalyst. Hydrogen peroxide aqueous solution (35%, w/w) was pumped into the extraction chamber and immediately decomposed by the catalyst. The infusion time of the hydrogen peroxide solution was 130 s (infusion starting 10 s before the start of MS data acquisition), and the MS data acquisition time was 120 s.

Proton-bound dimers (M_2_H^+^) are often more thermodynamically favored than protonated monomers (MH^+^), and clustering is enhanced by frequent collisions with drift gas, especially when trace moisture is present.^42^ In APCI, the protonated analyte ions readily undergo hydration and can form proton-bound dimers and larger clusters, with the extent of dimer formation dependent on molecular basicity and structure.^43^ In fact, dimer or adduct formation is a common phenomenon observed in IMS.^44^ Tozihi et al. combined IMS with *ab initio* calculations to show that the observed proton-bound dimers are stabilized by noncovalent interactions.^44^ This effort helps to explain why dimers can be more stable than isolated monomers under IMS conditions. Moreover, choosing the dimer instead of the monomer for optimization can be beneficial because dimers are often more sensitive to experimental conditions that affect ion–molecule clustering, such as analyte concentration, humidity, ionization efficiency, and drift gas composition. In IMS and APCI, proton-bound dimers react strongly to small changes in ion–molecule collision environments.^45^ Due to the above reasons, the ethyl acetate dimer peak intensity and its full width at half-maximum (fwhm) were used to determine the optimal parameters.

The dual ionization COME-IM (-QqQ-MS) operating parameters were optimized by adjusting the APCI corona needle and nESI voltages, drift-tube voltage, drift gas flow rate, nESI hydrodynamic pump pressure, and pulse widths of both ion gates (Figure 2). The APCI voltage, nESI voltage, drift-tube voltage, nESI pump pressure, and first ion gate pulse width were optimized on a standalone IM separation cell (Figure 2A–J). The second ion gate pulse width and drift gas flow rate were optimized on the IM-MS setup (Figure 2K–N). The drift-tube voltage, drift gas flow rate, first ion gate pulse width, and second ion gate pulse width were optimized in the APCI mode. On the other hand, the nESI voltage was optimized in the SESI mode. The time sequences for the standalone IMS and online IM-MS are presented in Figure S10A–C.

**Figure 2:**
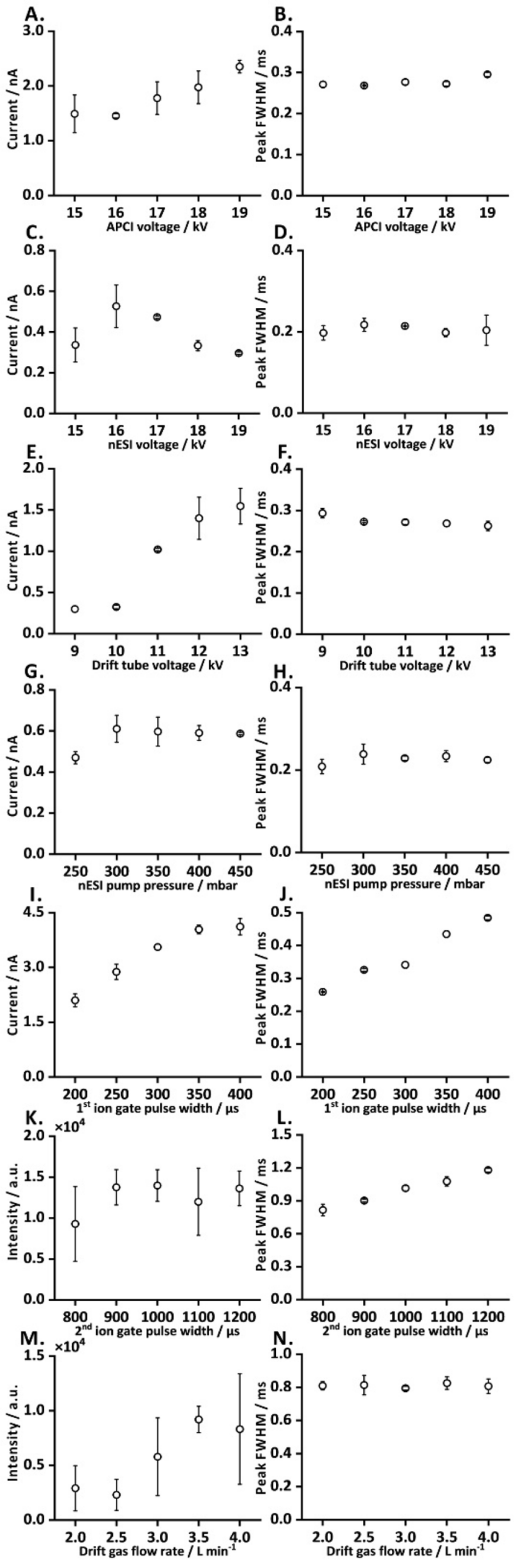
Optimization of key parameters of the dual ionization COME-IM­(-QqQ-MS) system. Parameters optimized using the standalone ion-mobility separation cell: (A) APCI voltage—peak intensity; (B) APCI voltage—fwhm; (C) nESI voltage—peak intensity; (D) nESI voltage—fwhm; (E) drift-tube voltage—peak intensity; (F) drift-tube voltage—fwhm; (G) nESI pump pressure—peak intensity; (H) nESI pump pressure—fwhm; (I) first ion gate pulse width—peak intensity; (J) first ion gate pulse width—fwhm. Parameters optimized using the online IM-MS setup: (K) second ion gate pulse width—peak intensity; (L) second ion gate pulse width—fwhm; (M) drift gas flow rate—peak intensity; (N) drift gas flow rate—fwhm. Please note that only the last two rows are related to IM-MS, while the first five rows are related to standalone IMS.

To optimize ion source parameters, the output voltages for APCI and nESI were in the range of 15 to 19 kV, remaining below the power supply’s maximum limit of 20 kV. Optimal performance was observed at 19 kV for APCI voltage and 16 kV for nESI voltage, which yielded the highest peak intensities with comparable fwhm (Figure 2A–D). The drift-tube voltage was optimized up to a maximum of 13 kV, beyond which an electric field instability was observed. A voltage of 13 kV was selected for subsequent measurements, as it provided a greater signal intensity and a consistent fwhm (Figure 2E, F). Furthermore, sample peak intensity and shape were evaluated across different drift-tube voltages to assess their influence on signal quality (Figure S11). Following the optimization, the nESI pump pressure and drift gas flow rate were adjusted to 450 mbar and 3.5 L min^–1^, respectively, to achieve a high peak intensity with low standard deviation and consistent fwhm (Figure 2G, H, M, N). The first ion gate pulse width was optimized in the range of 200 to 400 μs by evaluating the peak intensity and fwhm of the sample peak. A pulse width of 300 μs was selected to reach a balance between the peak resolution and signal intensity (Figure 2I, J). Likewise, the second ion gate pulse width was optimized over a range of 800 to 1200 μs, with distinct peak separation observed at 800 μs. Based on the lowest fwhm value, an optimal pulse width of 800 μs was chosen to enable effective separation of ethyl acetate monomer and dimer peaks in the SIM-IM spectra (Figure 2K, L). The optimized experimental parameters are listed in Table S2.

The nESI electrolyte composition for the analysis of ethyl acetate and nicotine was optimized using five different methanol–water mixtures, each containing 5% (v/v) acetic acid. For ethyl acetate, a 35% (v/v) aqueous methanol solution with 5% acetic acid provided the highest dimer signal intensity while retaining an acceptable monomer signal intensity (Figure S12A). In contrast, nicotine analysis revealed optimal dimer signal intensity with an 80% (v/v) aqueous methanol solution containing 5% (v/v) acetic acid, also yielding a satisfactory monomer signal performance (Figure S12B). Therefore, different nESI electrolytes were used for the two analytes (cf. Figure S6B, C).

### Analysis of VOCs Using Dual Ionization IM-MS Incorporating APCI and SESI

A dual ion source combines APCI and SESI for analyzing different physicochemical properties of VOCs, such as nicotine and ethyl acetate. Nicotine—a polar, basic compound with high gas-phase proton affinity^46,47^ —ionizes efficiently in SESI due to its moderate volatility and strong gas-phase basicity; thus, it is highly responsive to soft, gas-phase proton transfer. In contrast, ethyl acetate is a moderately polar, weakly basic ester with high volatility,^48^ making it more amenable to ionization by APCI, where a corona discharge enables chemical ionization through charge transfer in the solvent vapor phase. By integration of both APCI and SESI into a single setup, this dual ion source design allows for the optimized detection of highly polar, gas-phase basic VOCs as well as moderately polar VOCs, ensuring broad chemical coverage and enhanced analytical performance.

Two VOCs with distinct physicochemical properties—nicotine (1.5 × 10^–1^ M nicotine, before dilution) and ethyl acetate (1.5 × 10^–2^ M ethyl acetate, before dilution)—were analyzed using the two ionization modes available in the COME-IM-QqQ-MS setup. Please note that selected reaction monitoring (SRM) or multiple reaction monitoring may offer higher selectivity and potentially better sensitivity. However, in this proof-of-concept study, we aimed to keep the experimental design as simple as possible; therefore, we used the SIM mode. At a nicotine concentration of 1.5 × 10^–1^ M, peaks for nicotine monomer (APCI source; drift time: 7.99 ms; resolving power, *R*_p_ = 9.6) and dimer (APCI source; drift time: 12.43 ms; resolving power, *R*_p_ = 14.9) were observed. Note that all drift times were measured from the peak maximum. The reduced mobility values were determined to be 1.61 cm^2^ V^–1^ s^–1^ for nicotine monomer and 1.07 cm^2^ V^–1^ s^–1^ for nicotine dimer, respectively (Figure 3A, B), consistent with the reference values reported in the literature (1.53 cm^2^ V^–1^ s^–1^ and 1.08 cm^2^ V^–1^ s^–1^ for nicotine monomer and dimer, respectively^4^). For ethyl acetate (1.5 × 10^–2^ M), the monomer (APCI source; drift time: 6.66 ms; resolving power, *R*_p_ = 8.1) and dimer (APCI source; drift time: 8.21 ms; resolving power, *R*_p_ = 9.9) peaks were recorded. The corresponding *K*_0_ values are 1.84 cm^2^ V^–1^ s^–1^ and 1.54 cm^2^ V^–1^ s^–1^ for the monomer and dimer (cf. Figure 3C, D). According to the literature, *K*_0_ values are 1.92 cm^2^ V^–1^ s^–1^ for ethyl acetate monomer and 1.59 cm^2^ V^–1^ s^–1^ for ethyl acetate dimer,^49^ or 1.94 cm^2^ V^–1^ s^–1^ for ethyl acetate monomer and 1.53 cm^2^ V^–1^ s^–1^ for ethyl acetate dimer.^50^ In the SESI mode, the nicotine monomer exhibited ∼2× higher signal intensity, while the nicotine dimer showed an increase of ∼17×. Conversely, the APCI mode yielded ∼2× higher signal intensity for the ethyl acetate monomer and ∼5× higher intensity for the dimer, enhancing detection of the target analyte.

**Figure 3:**
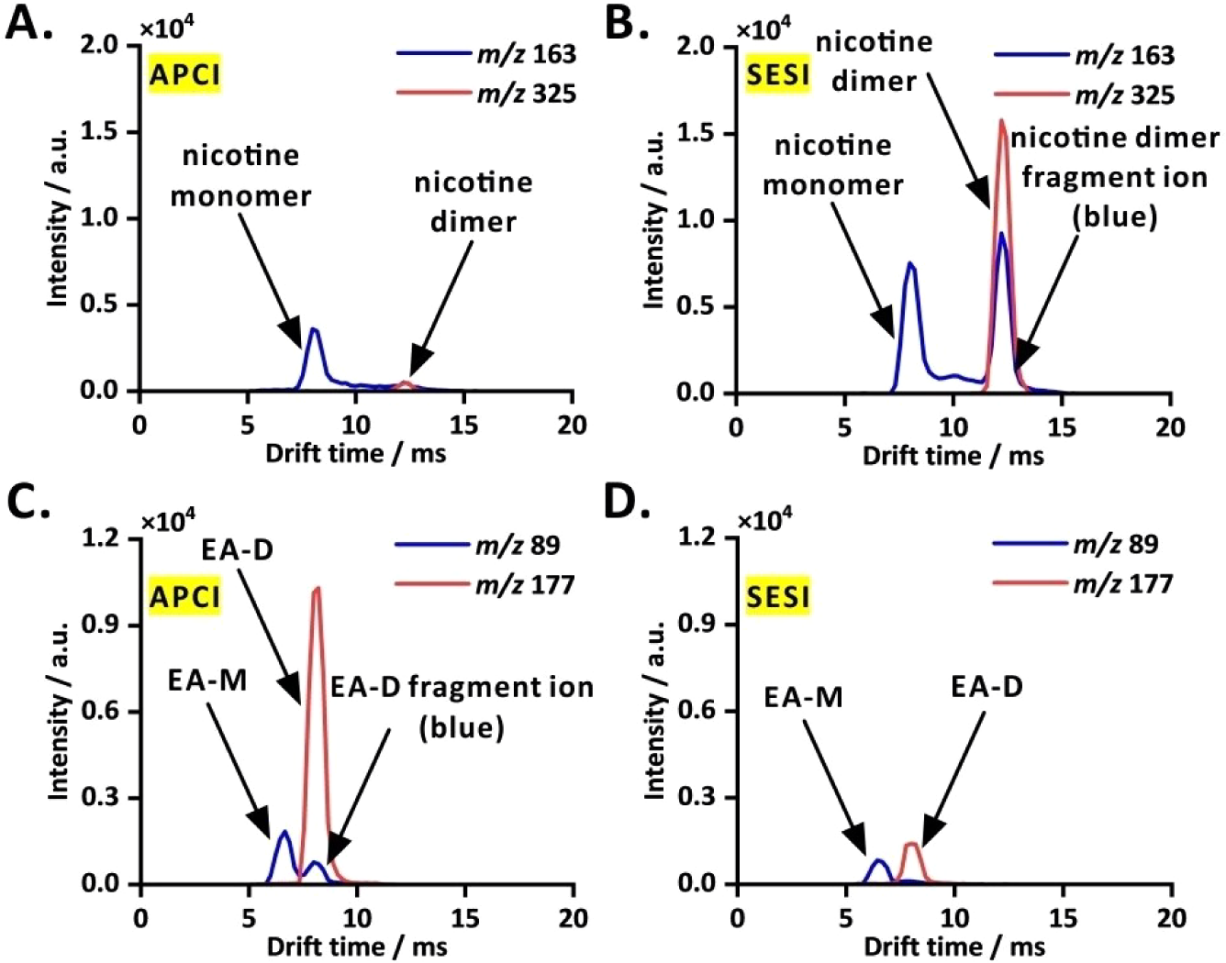
Dual ionization COME-IM-QqQ-MS performance in analyses of high-polarity and medium-polarity VOCs. (A) A polar compound (1.5 × 10^–1^ M nicotine, before dilution) in APCI mode; (B) nicotine in SESI mode; (C) a moderately polar compound (1.5 × 10^–2^ M ethyl acetate, before dilution) in APCI mode; (D) ethyl acetate in SESI mode. EA-M, ethyl acetate monomer; EA-D, ethyl acetate dimer.

In the SIM-mode SESI-IM spectrum of nicotine, an additional peak was observed at 12.21 ms in *m*/*z* channel 163 (Figure 3B). This peak was confirmed by tracking the protonated nicotine monomer and dimer (*m*/*z* = 163 and 325) in a single run. The additional peak was identified as a partial dissociation fragment ion originating from the nicotine dimer (cf. refs 51, 52 ). Furthermore, an additional peak at 7.99 ms in *m*/*z* channel 89 was seen when examining the APCI-IM spectra of protonated ethyl acetate monomer and dimer (*m*/*z* 89 and 177; Figure 3C). When ionization methods were compared, SESI-IM spectra of nicotine monomer (*m*/*z* 163; Figure 3B) showed characteristic fragment ions. In contrast, APCI-IM spectra of ethyl acetate monomer (*m*/*z* 89; Figure 3C) revealed a smaller fraction of dissociated ethyl acetate dimer and a distinct peak with a long drift time. Due to ion packets being transferred from the drift tube to the MS inlet in order of mobility, beginning with high mobility and followed by low mobility, the higher-mobility monomer ion peak appeared first. Subsequently, the dimer ions with a lower mobility entered the MS inlet and were partially dissociated into monomer ions.

### Real Sample VOC Analysis Using Dual Ionization COME-IM-QqQ-MS

Millet wine is a traditional fermented beverage of Taiwanese indigenous harvest festivals.^53^ The characteristic fruity aroma of millet wine is largely attributed to VOCs formed during fermentation, with ethyl acetate serving as a principal contributor to its distinctive flavor profile.^54^ Similarly, ethyl acetate is also present in whiskey contributing to its fruity aroma.^49,55^ The dual ionization COME-IM-QqQ-MS system was employed to analyze the VOC profiles of millet wine and whiskey without diluting the liquor samples. In SIM mode under positive ionization, two *m*/*z* values (89 and 177) were monitored for the protonated ethyl acetate monomer and dimer. As expected, the IM spectra of millet wine and whiskey showed two distinct peaks corresponding to the monomer and dimer of ethyl acetate (Figure 4A, C).

**Figure 4:**
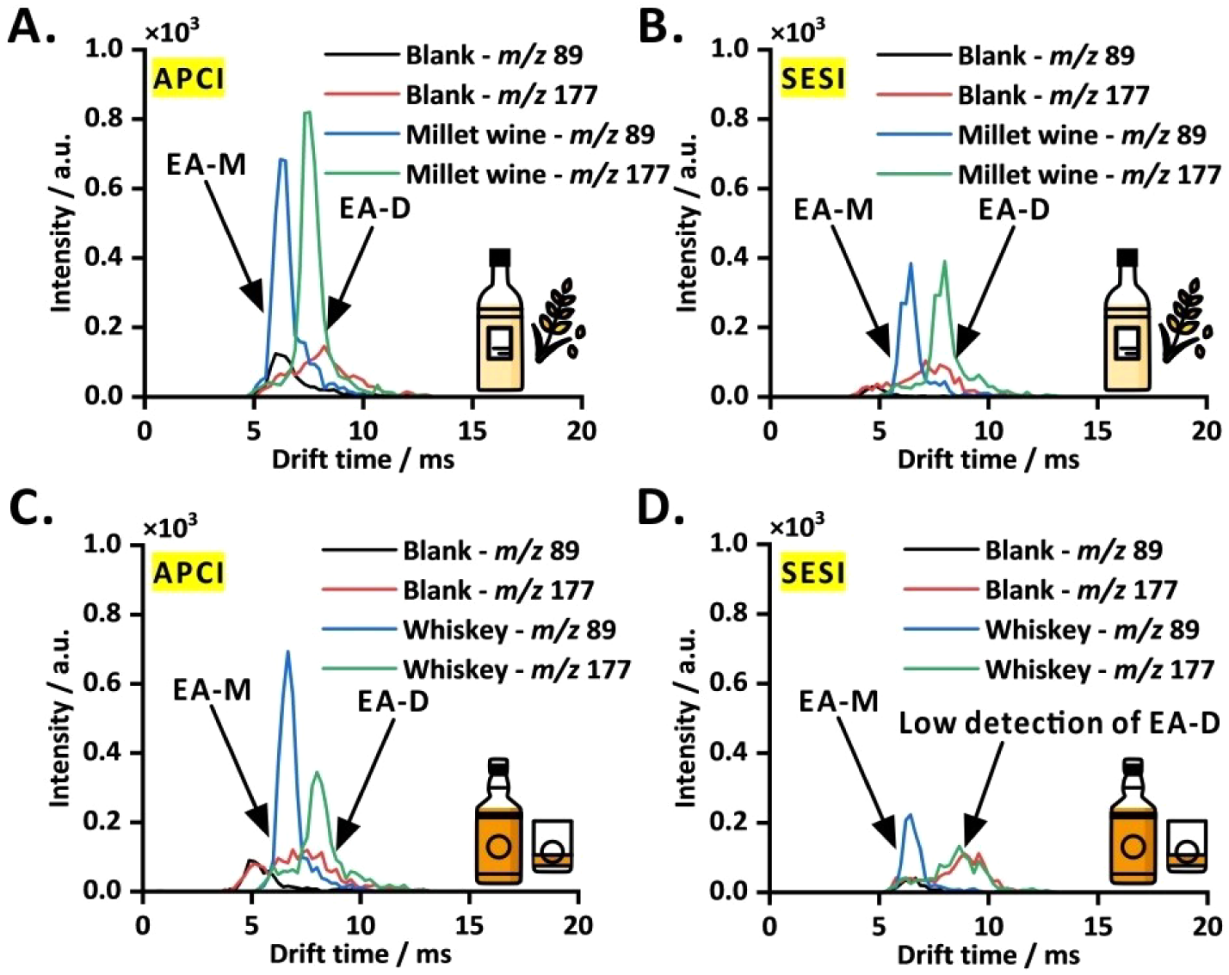
Analysis of VOCs in undiluted real samples (200 μL) by dual ionization COME-IM-QqQ-MS. (A) Millet wine in APCI mode; (B) millet wine in SESI mode; (C) whiskey in APCI mode; (D) whiskey in SESI mode. EA-M, ethyl acetate monomer; EA-D, ethyl acetate dimer.

Both ethyl acetate monomer and dimer peaks were detected in both ionization modes for the millet wine sample (Figure 4A, B) and whiskey sample (Figure 4C, D). As expected, they were lower in SESI mode than in APCI mode due to the differences in ionization efficiency. When analyzing the liquor samples, the carryover issue was observed in both ionization mode analyses. Ethanol vapor was used to remove residual ethyl acetate from the custom-designed extraction chamber cover and drift-tube connection (PTFE tubing). Despite that cleaning step, some ethyl acetate was retained in the system after sample analysis, contributing to moderate blank signals (Figure 4A–D). Although the detectability of the presented approach is in the millimolar range, complex food-related VOCs in liquid matrices can be analyzed without extensive sample preparation. Further improvements in this aspect can be made by incorporating drift-tube heaters and operating the mass spectrometer in SRM mode.

### Study of the Moisture Effect in IM Spectra

We examined the effect of the moisture trap on the peak intensity. The presence of moisture in the ion-mobility separation cell promotes the formation of hydrated ion clusters, which may decrease the abundance of protonated analyte species and suppress analyte signals.^56^ Due to this effect of residual water,^57–59^ molecular sieves are typically used to purify and remove the moisture from the drift gas for studies involving IMS. However, the COME step likely increases the moisture content in the ion source. It was previously shown that moisture in the drift gas effectively increases the ion’s physical size, creating more drag and significantly slowing down its travel through the drift tube.^60–62^ Consequently, a higher moisture level may lead to longer drift times and peak broadening, as the constant fluctuation between hydrated and dry states blurs the arrival time of the ions.

To further characterize the developed system, we investigated the influence of drift gas moisture on SIM-IM spectra in QqQ-MS by modifying the configuration of the drift gas molecular sieve tube to create high- and low-moisture conditions in the drift region. For high-moisture conditions, the molecular sieve tube was removed, allowing unprocessed nitrogen gas to be directly introduced into the drift tube. For low-moisture conditions in the ion-mobility separation cell, the molecular sieve tube was placed before the drift gas flow controller to remove moisture from the nitrogen supply. According to the results, moisture can significantly affect the peak intensity of the target analyte, potentially influencing the sensitivity of the IM-QqQ-MS measurements. The drift gas had higher moisture content after removing the molecular sieve tube, which led to a decrease in the ethyl acetate (1.5 × 10^–2^ M in 200 μL sample) peak intensity in all three replicates in APCI mode (Figure S13A, B). To further confirm the signal decrease of the sample peak in SIM-IM spectra, the moisture effect on nicotine (1.5 × 10^–1^ M in 200 μL sample) was analyzed in SESI mode under the same conditions as using APCI mode to investigate the moisture effect on nicotine. When the molecular sieve tube was removed from the drift gas supply, it showed a sample peak signal decrease for both nicotine monomer and dimer (Figure S14A, B). Based on the average peak intensity (calculated based on three replicates) for ethyl acetate in APCI-IM-MS analysis, the removal of the molecular sieve tube resulted in an over 2-fold decrease in ethyl acetate monomer and a 3-fold decrease in dimer signal intensity (Figure S13C). For nicotine SESI-IM-MS analysis, removing the molecular sieve tube led to a minor signal decrease for nicotine monomer and almost a 2-fold decrease for nicotine dimer (Figure S14C).

### Drift Time and Intensity Correction with Isotopically Labeled Ethyl Acetate in IM Spectra

Isotopically labeled internal standards are widely used in MS analyses.^63,64^ Similarly, drift time correction in IMS has been applied in studies involving varying temperature conditions.^65^ When operating the drift-tube IM-MS prototype, we observed variations in drift times and peak intensities (Figure 5A), which may have resulted from space-charge effects, gas-flow inhomogeneities, and fluctuations in temperature and pressure. To improve the repeatability of SIM-mode IM data, 1,2-^13^C_2_-labeled ethyl acetate (^13^CH_3_^13^CO_2_C_2_H_5_) was employed as an internal standard for both drift time and intensity correction. Drift time and intensity corrections were performed by mixing 100 μL of 1.5 × 10^–2^ M ethyl acetate (analyte) with 100 μL of 1.5 × 10^–2^ M isotopically labeled ethyl acetate (internal standard). The first replicate was designated as the “benchmark”, and correction ratios for subsequent replicates were calculated by dividing the drift time and intensity values of the protonated isotopically labeled ethyl acetate dimer (*m*/*z* 181) in the benchmark by those in the replicates 2–10 (*m*/*z* 181). These correction ratios were then applied to the analyte signals (*m*/*z* 177) to obtain normalized drift times and intensities, thereby reducing the run-to-run variability (Figure 5B).

**Figure 5:**
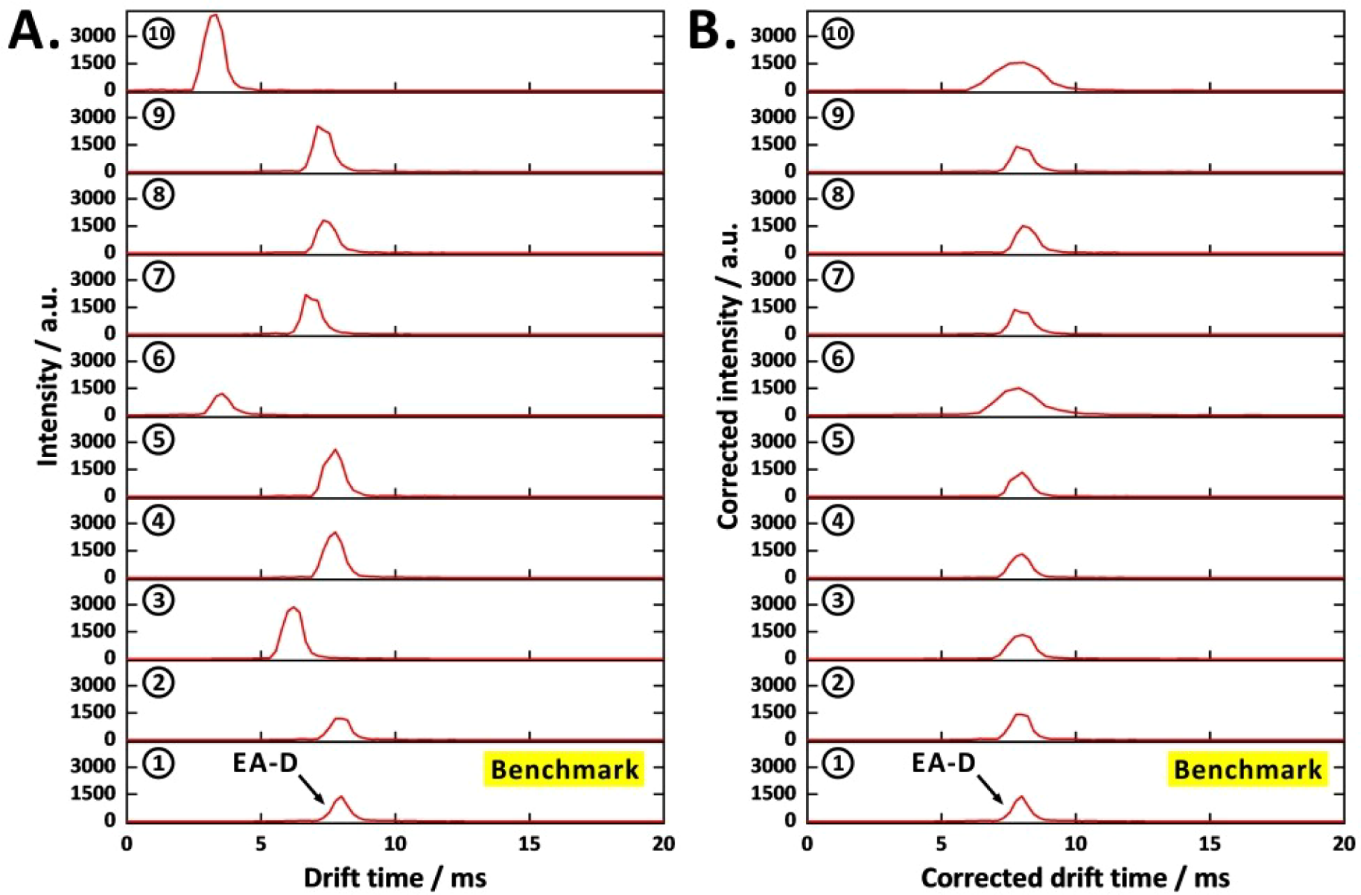
Drift time and intensity correction of SIM-mode IM spectra (*m*/*z* 177) of ethyl acetate dimer via isotopically labeled internal standard in a repeatability test: (A) before correction and (B) after correction. EA-D, ethyl acetate dimer. APCI voltage, 19 kV. Before analysis, 100 μL of 1.5 × 10^–2^ M ethyl acetate and 100 μL of 1.5 × 10^–2^ M isotopically labeled ethyl acetate (before dilution) were mixed with 2 mL of water and 100 mg of manganese­(IV) oxide. Four events were configured in SIM mode (event time, 0.412 s).

The use of the isotopically labeled internal standard significantly improved IM peak alignment in both drift time and signal intensity (Figure 5B). The corrected drift times were tightly clustered, and peak intensities were consistent across replicates, confirming successful normalization. The relative standard deviation (RSD) of ethyl acetate dimer intensity decreased from 40.2% to 7.8% for 10 replicates, while the RSD of drift time dropped from 25.2% to 0.6%. The same approach was applied to the ethyl acetate monomer IM spectra to achieve peak alignment (Figure S15). For the monomer peak, the RSD of intensity was reduced from 15.8% to 8.4%, and the RSD of drift time decreased from 33.3% to 1.9%. However, for strongly drift time-shifted peaks in the IM spectra, the corrected peak shapes displayed a lower resolution than those with minimal drift time shifts. Overall, these results confirm the effectiveness of using isotopically labeled internal standards to compensate for run-to-run variability in drift-tube IM-MS analyses, thereby enhancing data reliability.

## Conclusions

The main contribution of this study is the integration of a dual ionization IM-QqQ-MS system with environmentally friendly COME for online VOC analyte introduction. The system is operated using a custom-developed GUI. Integration of dual ionization with COME-IM-QqQ-MS enables direct, apple-to-apple comparisons between ionization modes, facilitating the analysis of VOCs with diverse physicochemical properties, such as nicotine (a polar, basic compound with high gas-phase proton affinity) and ethyl acetate (a moderately polar, weakly basic ester with high volatility). This house-built platform successfully differentiated monomeric and dimeric species of both VOCs based on their unique drift times and reduced mobilities, with SESI providing higher sensitivity for nicotine and APCI showing enhanced detection of ethyl acetate. The presence of moisture in the ionization region and drift region was shown to suppress the ion signal intensities, pointing to the critical role of moisture control in accurate and reliable IMS measurements. An isotopically labeled internal standard corrected drift time and intensity variations, enabling the alignment of SIM-IM spectra. In the future, the dual ionization setup may be expanded to include alternative ion sources, such as an X-ray^17^ or UV photoionization source,^66^ for comparative assessment of ionization performance. Additionally, it would be of interest to distinguish the structural isomers of the analyzed VOCs. However, achieving this goal hinges on a significant improvement in the resolving power, which requires further instrumental developments.

## Supplementary Material





## Notes

Data processing of IM spectra with QqQ-MS; additional tables (S1, S2); additional figures (S1–S15) (PDF)
Video (S1) (MP4)
