# Supplementary material for: Dual Ionization Ion-Mobility Mass Spectrometry Hyphenated with Catalytic Oxygenation-Mediated Extraction
Source: ACS Meas Sci Au. 2026 Jan 2;6(1):204–13. doi: 10.1021/acsmeasuresciau.5c00160 (PMC12921609; doi:10.1021/acsmeasuresciau.5c00160)
Supplement: Supplementary file 1 [file tg5c00160_si_001.pdf]

SUPPORTING INFORMATION

Dual Ionization Ion-Mobility Mass Spectrometry Hyphenated with Catalytic Oxygenation-Mediated Extraction

Tzu-Ching Tsai, Chamarthi Maheswar Raju, Pawel L. Urban*

Department of Chemistry, National Tsing Hua University

101, Section 2, Kuang-Fu Rd., Hsinchu, 300044, Taiwan

* Corresponding author:

P.L. Urban (urban@mx.nthu.edu.tw)

Data processing of IM spectra with QqQ-MS

The QqQ-MS data acquisition system was used to export chromatograms as ASCII files and then generate the ion-mobility spectrum of the IM-QqQ-MS. Data processing and calculations were performed using Excel (version 2019 Professional Plus; Microsoft, Redmond, WA, USA). When the second ion gate pulse period was set to 40 ms, the IM-QqQ-MS system collected data during 2 s corresponding to a drift time interval of 0.222 ms. In SIM mode, with the two events configured (event time, 0.206 s), the exported ASCII files contain 874 data points for a 180 s run time. Subsequently, the IM-QqQ-MS data—acquired at 2 s intervals—were averaged over the range of 9.71 data points aligned with 0.222 ms in the drift time scale. The average datasets were imported into OriginPro software (version 2025; Northampton, MA, USA) to plot the ion-mobility spectra based on the IM-QqQ-MS data. In the GUI, the ion-mobility spectra were plotted using the Matplotlib package in Python (version 3.8.10; Python Software Foundation, Wilmington, DE, USA). A 16-bit analog-to-digital converter, ADS1115 (Centenary Materials) converts analog output values from the IM-QqQ-MS into millivolts using a predefined reference voltage (±4.096 V) and a 16-bit resolution scaling factor, storing both the analog values and their corresponding timestamps relative to the analysis start time to facilitate the plotting of IM spectra immediately after their acquisition in the GUI.

ADDITIONAL TABLES

Table S1. Triple quadrupole mass spectrometer operating parameters.

Parameter	Unit	Value
Drying gas and nebulizing gas	-	nitrogen
Drying gas flow rate	L min^{-1}	3.0
Nebulizing gas flow rate	L min^{-1}	0.5
Desolvation line temperature	°C	50
Heat block temperature	°C	50

Table S2. Dual ionization COME-IM-QqQ-MS operating parameters.

Parameter	Unit	Value
Drift-tube temperature	°C	ambient
Drift-tube pressure	Torr	ambient
APCI voltage	kV	19
nESI voltage	kV	16
Drift-tube voltage	kV	13
nESI pump pressure	mbar	450
Drift gas	-	nitrogen
Drift gas flow rate	L min^{-1}	3.5
1st ion gate pulse width	µs	300
2nd ion gate pulse width	µs	800
Drift-tube length	cm	20
Drift region length	cm	12
Drift region electric field strength	V cm^{-1}	860

ADDITIONAL FIGURES

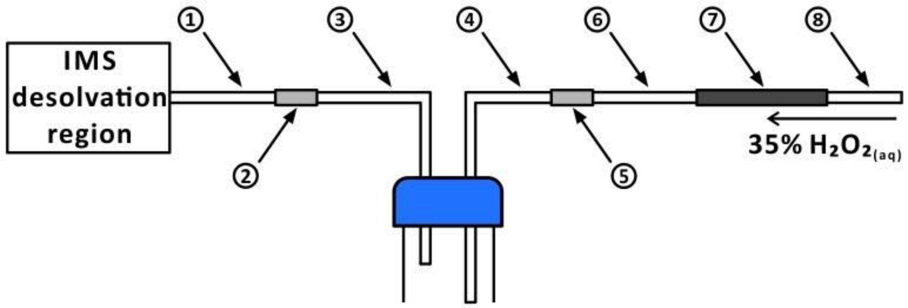

Figure S1. Extraction chamber tubing connections. (1) PTFE tubing (length, 250 mm; ID, 0.9 mm; OD, 1.59 mm); (2) silicone tubing (length, 18 mm; ID, 1.0 mm; OD, 2.0 mm); (3) PTFE tubing (length, 250 mm; ID, 0.8 mm; OD, 1.59 mm); (4) PTFE tubing (length, 200 mm; ID, 0.5 mm; OD, 1.59 mm); (5) silicone tubing (length, 18 mm; ID, 1.0 mm; OD, 2.0 mm); (6) PTFE tubing (length, 950 mm; ID, 0.3 mm; OD, 1.59 mm); (7) Tygon tubing (length, 300 mm; ID, 1.59 mm; OD, 3.18 mm); (8) PTFE tubing (length, 80 mm; ID, 0.8 mm; OD, 1.59 mm).

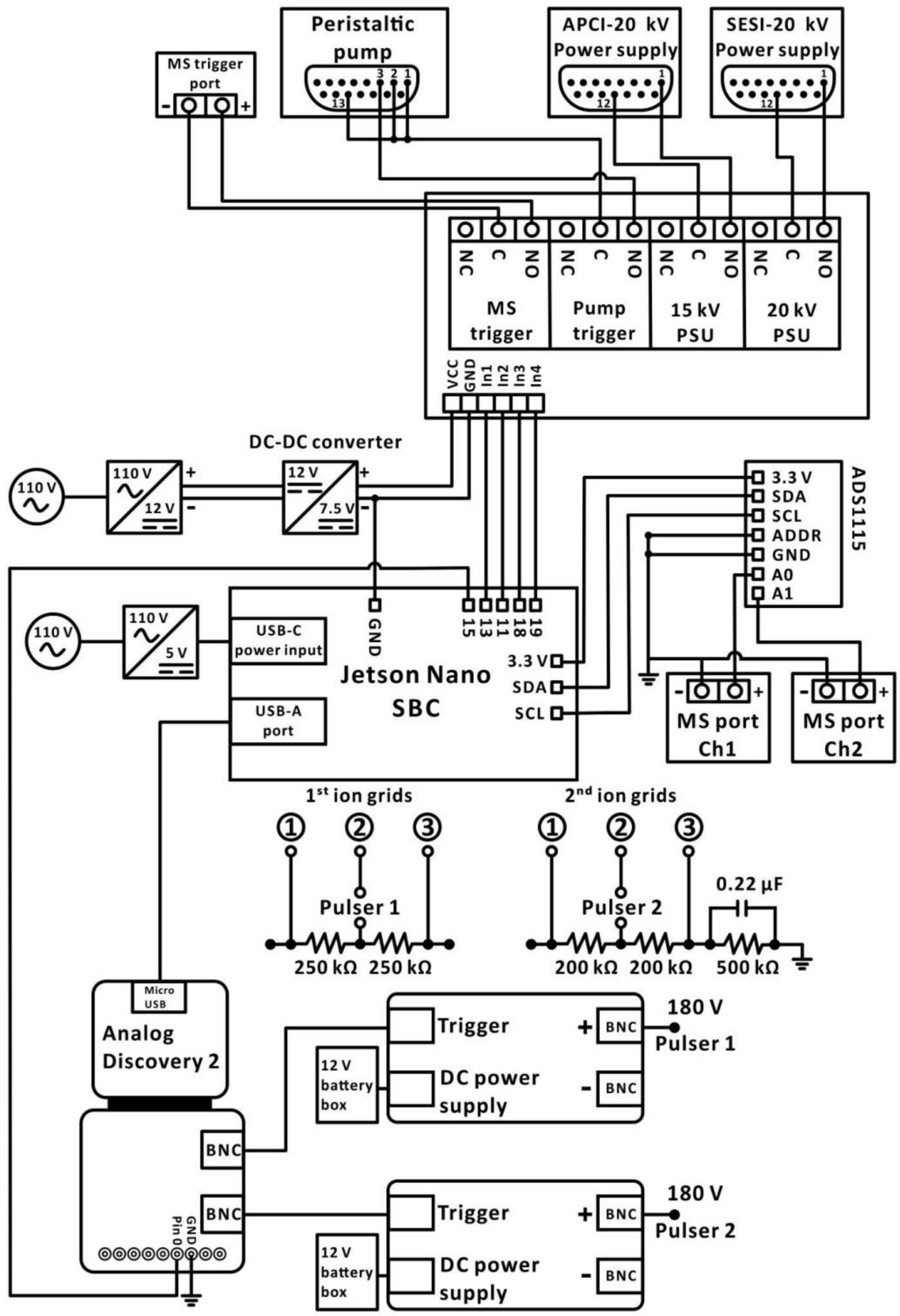

Figure S2. Electronic circuit diagram of dual ionization COME-IM-QqQ-MS control unit.

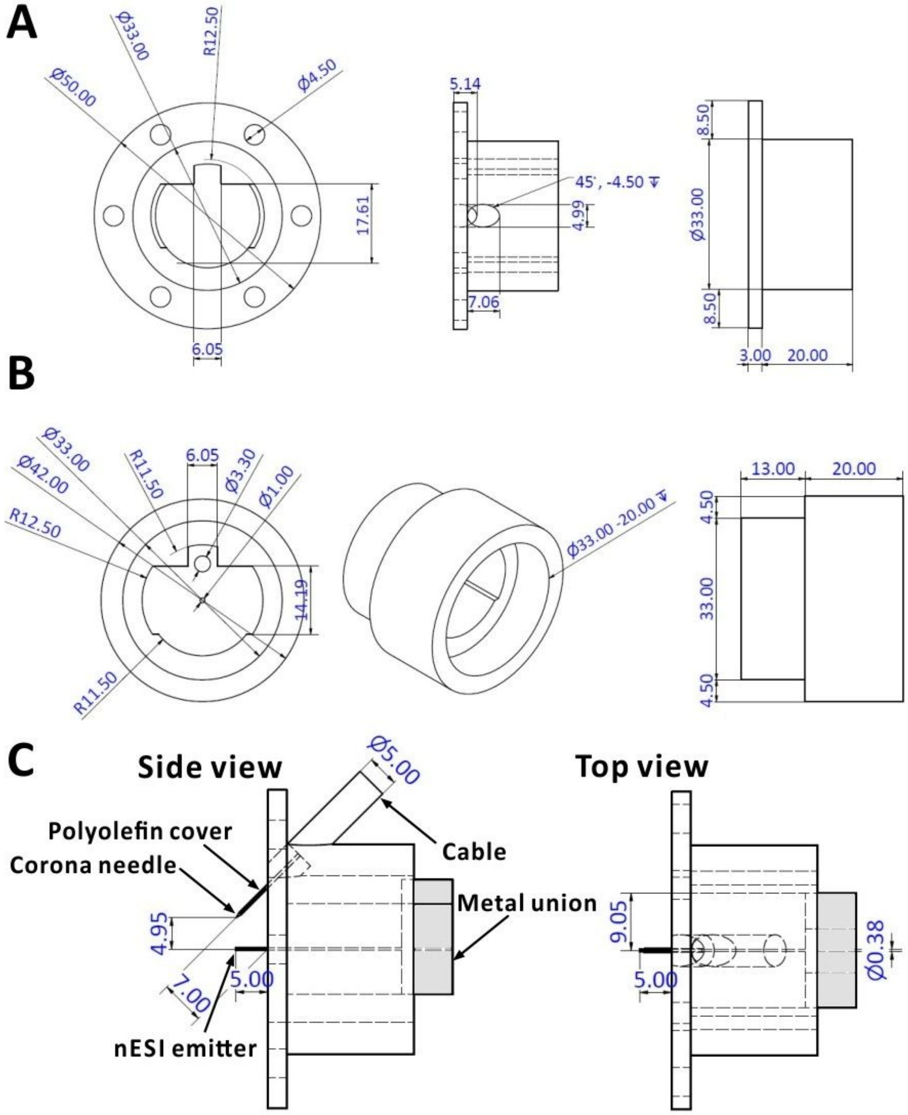

Figure S3. 3D-printed dual ion source holders: (A) front section; (B) rear section; (C) schematic of dual ion source alignment (units: mm).

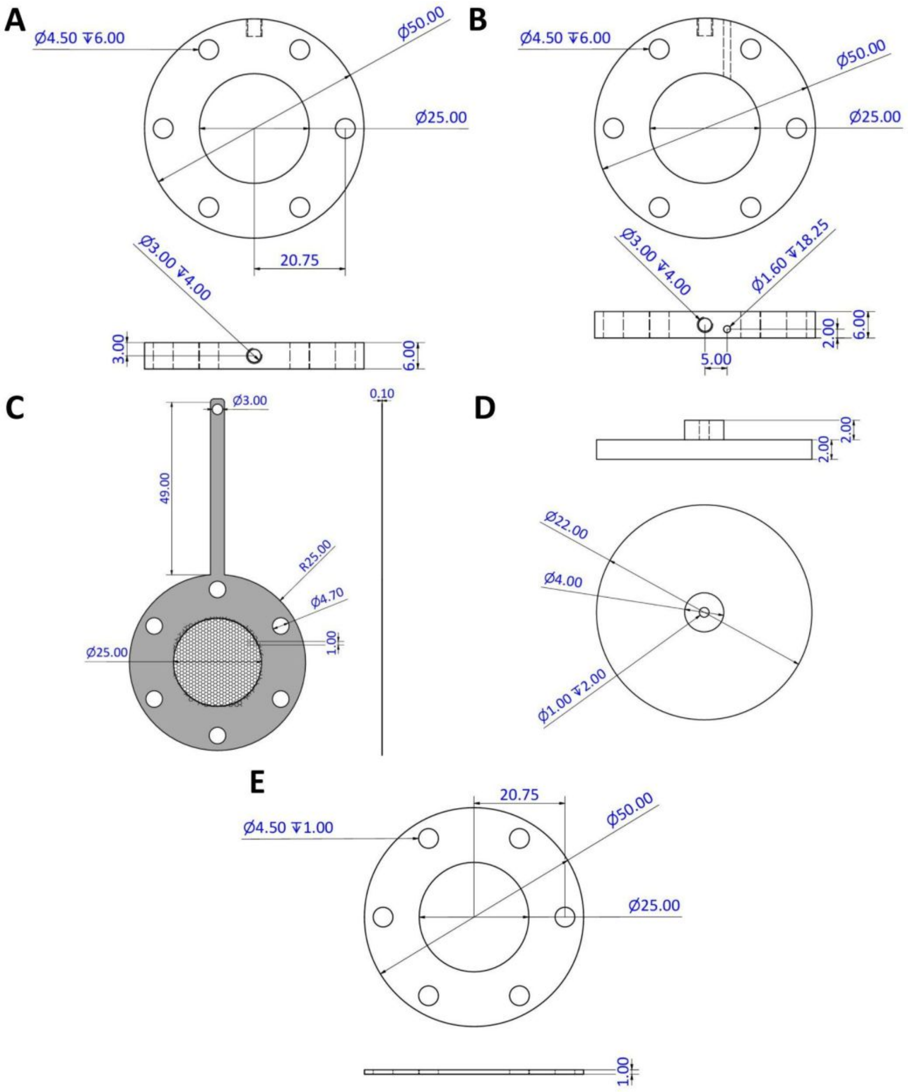

Figure S4. Drift-tube building elements: (A) stainless steel ring electrode; (B) sample injection ring electrode; (C) ion gate electrode; (D) Faraday plate; (E) ceramic spacer (unit: mm).

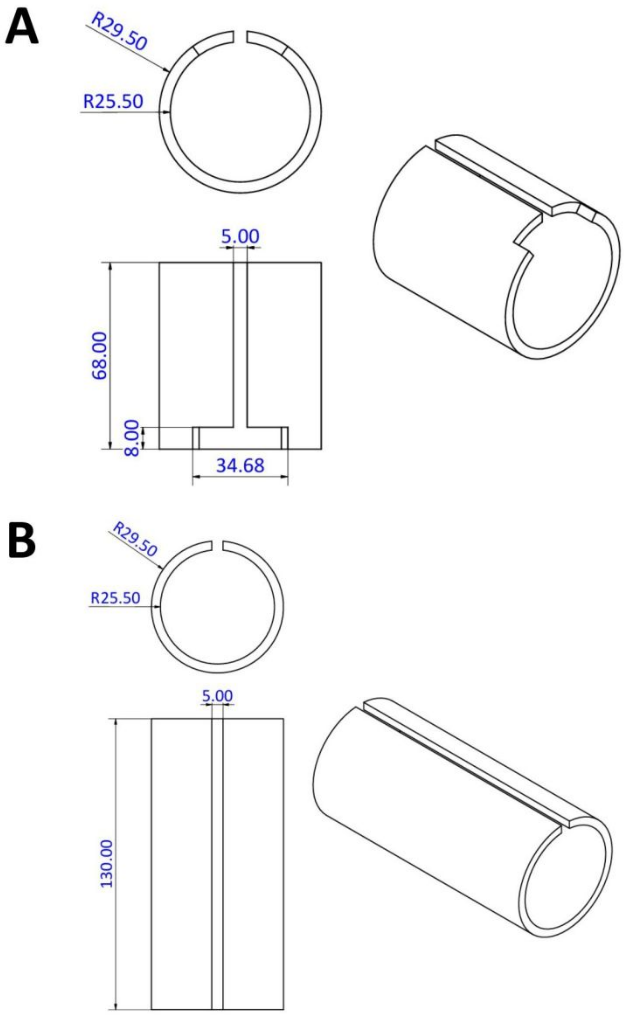

Figure S5. Drift-tube insulation elements: (A) front ceramic cover; (B) rear ceramic cover (unit: mm).

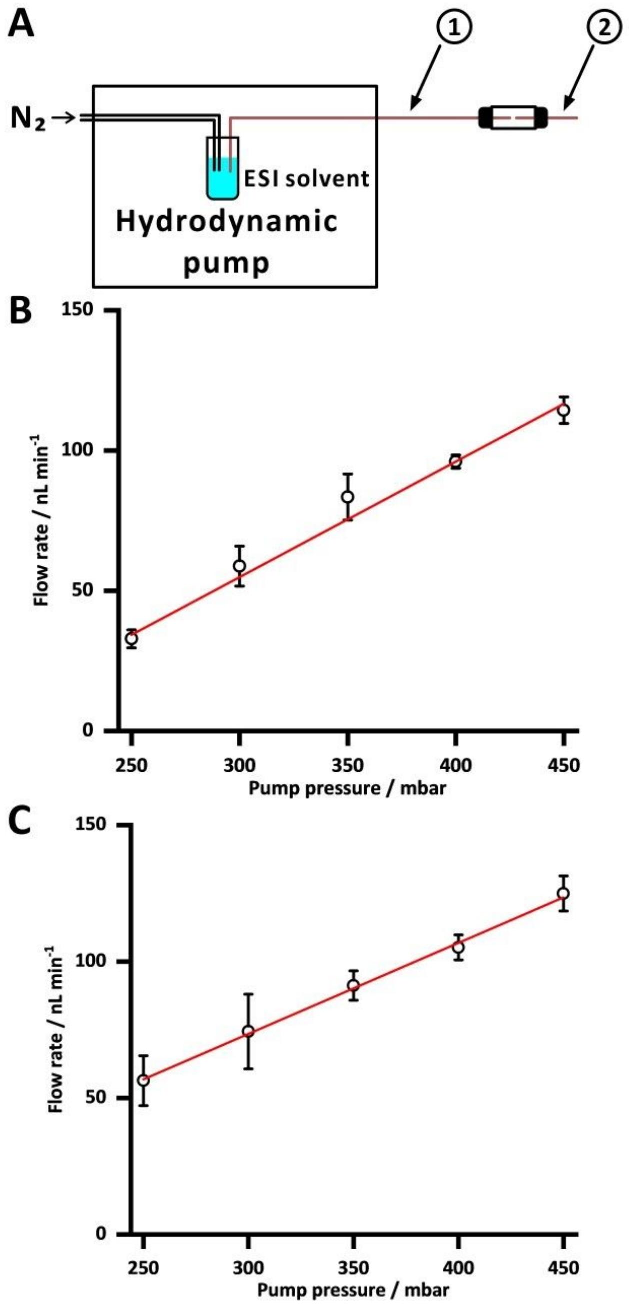

Figure S6. The relationship between liquid flow rate and hydrodynamic pump pressure in the nESI setup. No voltage was applied to the nESI emitter. (A) Schematic of the nESI emitter setup: (1) fused silica capillary (length, 450 mm; ID, 0.05 mm; OD, 0.375 mm); (2) fused silica capillary (length, 30 mm; ID, 0.02 mm; OD, 0.375 mm). (B) Calibration plot for 80% (v/v) aqueous methanol solution with 5% acetic acid. Calibration equation: *Flow rate* = (0.41 ± 0.02) *Pump pressure* + (-68.81 ± 6.26), R^2 = 0.993. Replicates, n = 3. (C) Calibration plot for 35% (v/v) aqueous methanol solution with 5% acetic acid. Calibration equation: *Flow rate* = (0.33 ± 0.01) *Pump pressure* + (-26.65 ± 5.21), R^2 = 0.993. Replicates, n = 3.

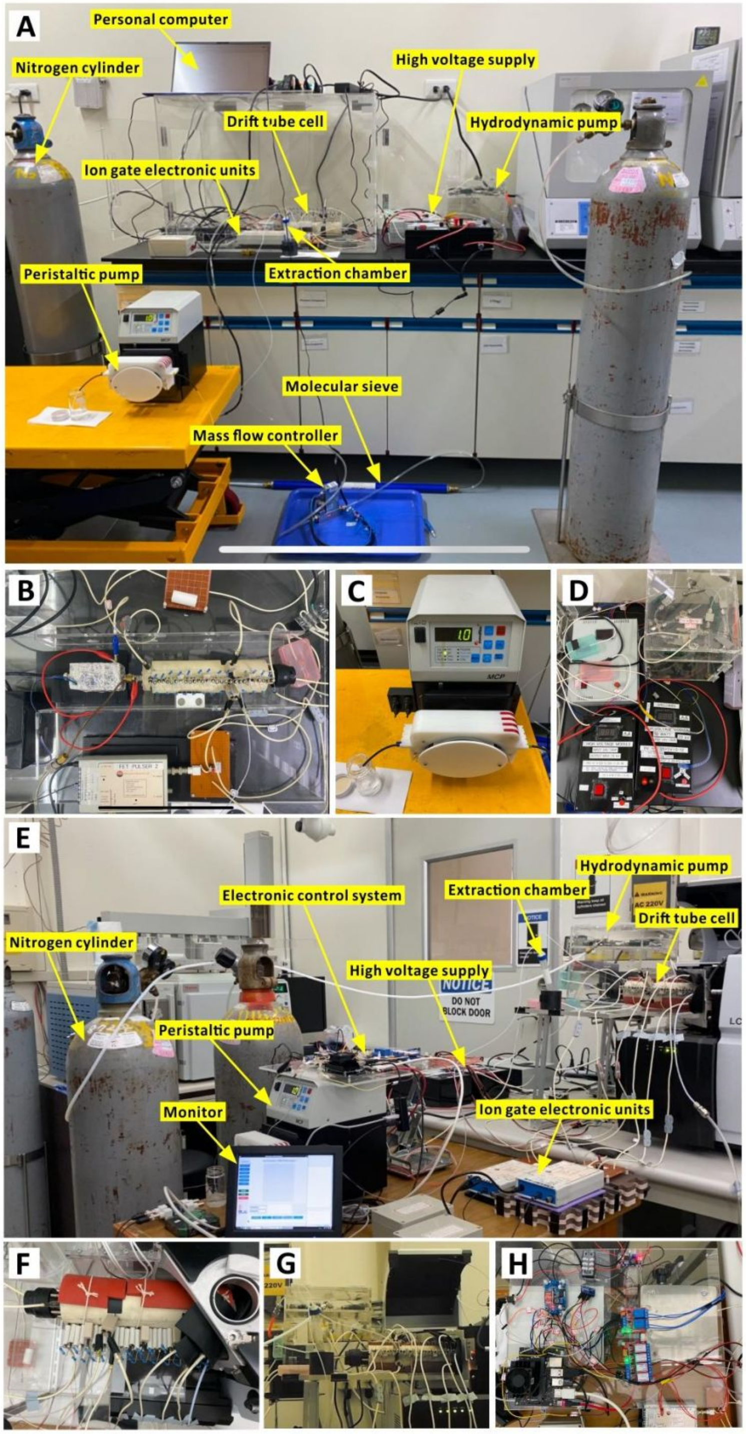

Figure S7. Photographs of the experimental setup: (A) offline standalone COME-IMS system overview; (B) top view of the setup; (C) peristaltic pump; (D) high-voltage power supply and hydrodynamic pump; (E) online dual ionization COME-IM-QqQ-MS system overview; (F) top view of drift-tube cell; (G) side view of drift-tube cell; (H) electronic control system.

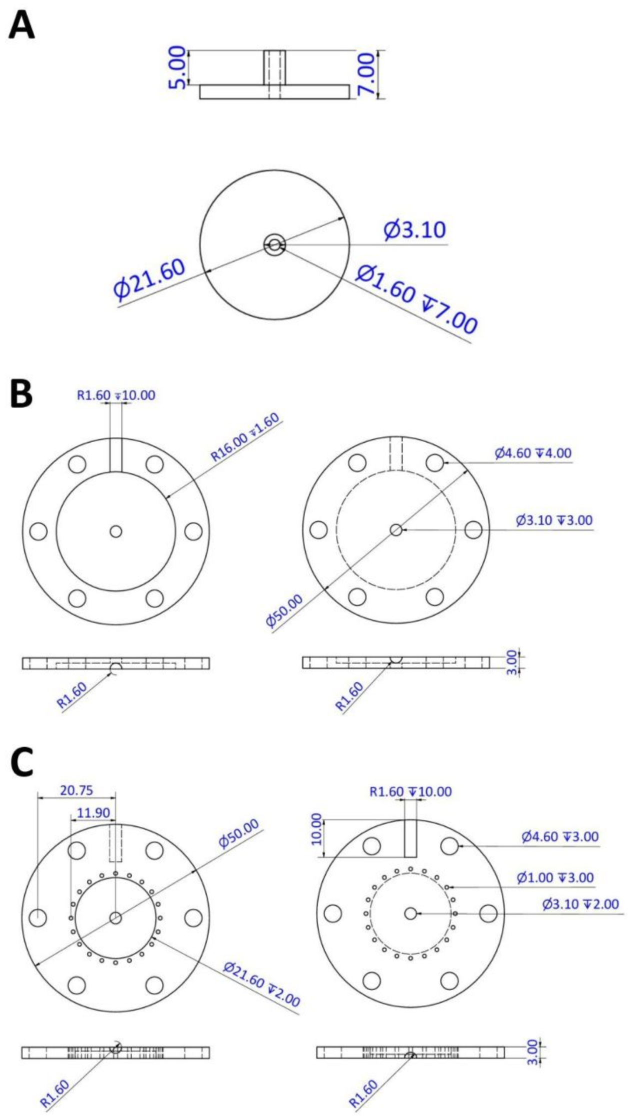

Figure S8. IM-QqQ-MS coupling elements. (A) Stainless steel ion transfer unit; (B) rear coupling plate; (C) front coupling plate (unit: mm).

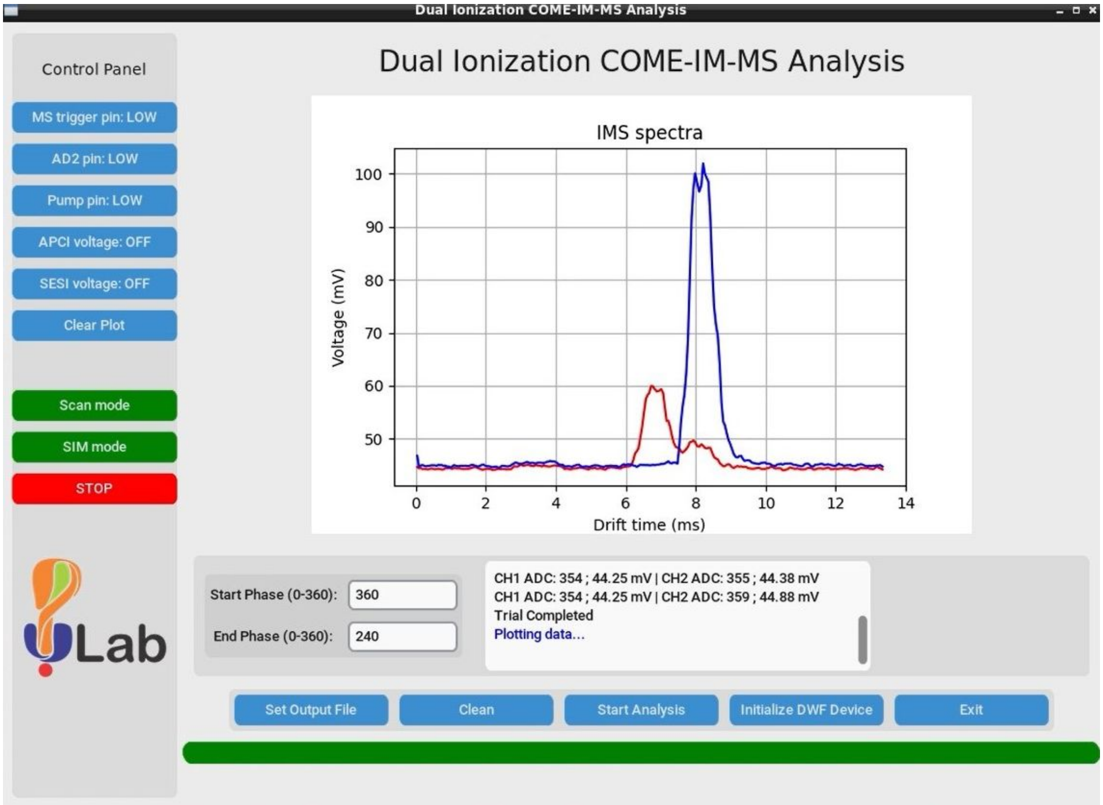

Figure S9. Screenshot of the analysis results from the dual ionization COME-IM-QqQ-MS automated control GUI. SIM-IM spectrum of ethyl acetate standard. MS analog output channel 1, *m/z* 89; MS analog output channel 2, *m/z* 177; ion gate start phase, 360°; ion gate end phase, 240°; MS run time, 2 min.

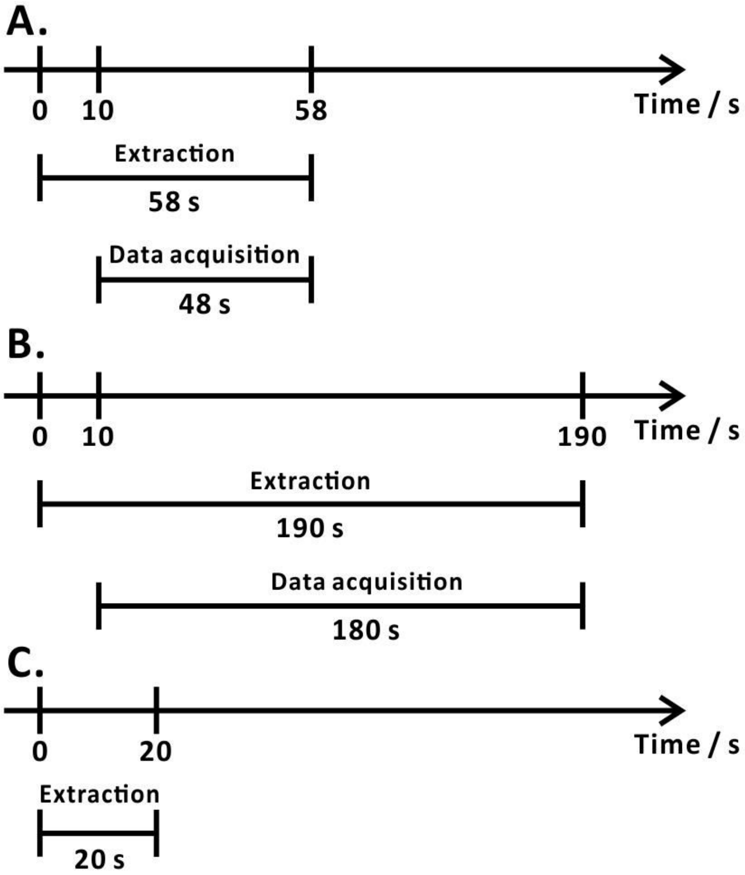

Figure S10. Dual ionization COME-IM-QqQ-MS and standalone COME-IMS analysis sequences. (A) Standalone COME-IMS analysis (data were collected in triplicate under stable signal conditions, with the third replicate selected as analysis result; average per spectrum: 200); (B) COME–IM–QqQ–MS analysis mode. The MS data acquisition time was set to 180 s, corresponding to a 0–20 ms ion-mobility spectrum.; (C) COME-IM-QqQ-MS cleaning mode (using 2 mL ethanol).

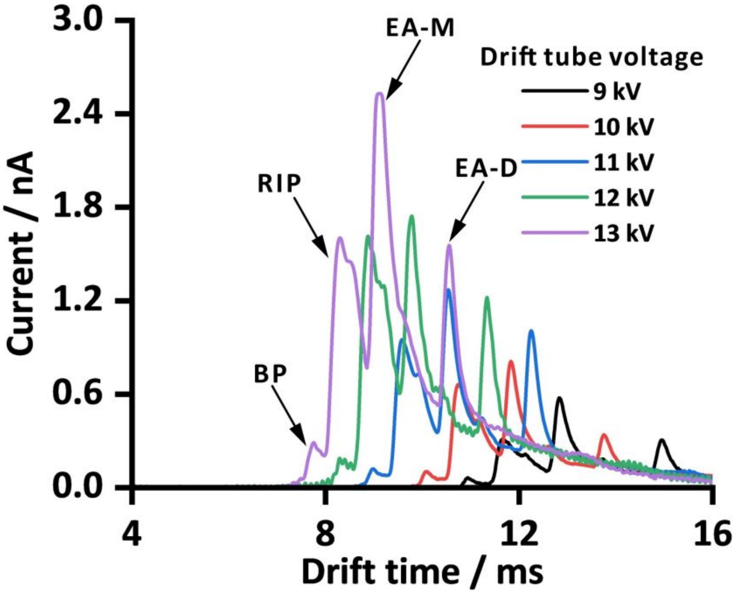

Figure S11. IM spectra of ethyl acetate monomer and dimer during drift-tube voltage optimization in the standalone APCI-COME-IMS setup. BP, blank peak; RIP, reactant ion peak; EA-M, ethyl acetate monomer; EA-D, ethyl acetate dimer. APCI voltage (for 13 kV drift tube voltage), 19 kV (6 kV potential difference was maintained between the ion source and the drift tube voltages); 200 µL of ethyl acetate at 1.5×10^{-2} M (before dilution) with 2 mL of water and 100 mg of manganese (IV) oxide.

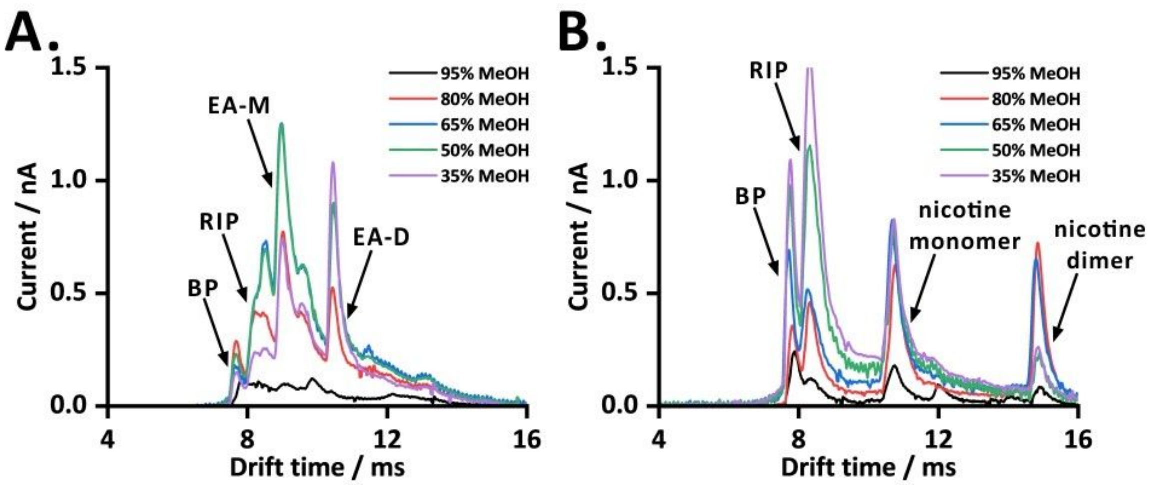

Figure S12. nESI electrolyte composition optimization on the standalone COME-IMS setup. (A) IM spectra revealing ethyl acetate monomer and dimer analyte peak distribution. BP, blank peak; RIP, reactant ion peak; EA-M, ethyl acetate monomer; EA-D, ethyl acetate dimer; (B) IM spectra revealing nicotine monomer and dimer analyte peaks distribution. BP, blank peak; RIP, reactant ion peak. APCI voltage, 19 kV; nESI voltage, 16 kV; drift tube voltage, 13 kV; 200 µL of ethyl acetate at 1.5×10^{-2} M (before dilution); 200 µL of nicotine at 1.5×10^{-1} M (before dilution) with 2 mL of water and 100 mg of manganese (IV) oxide.

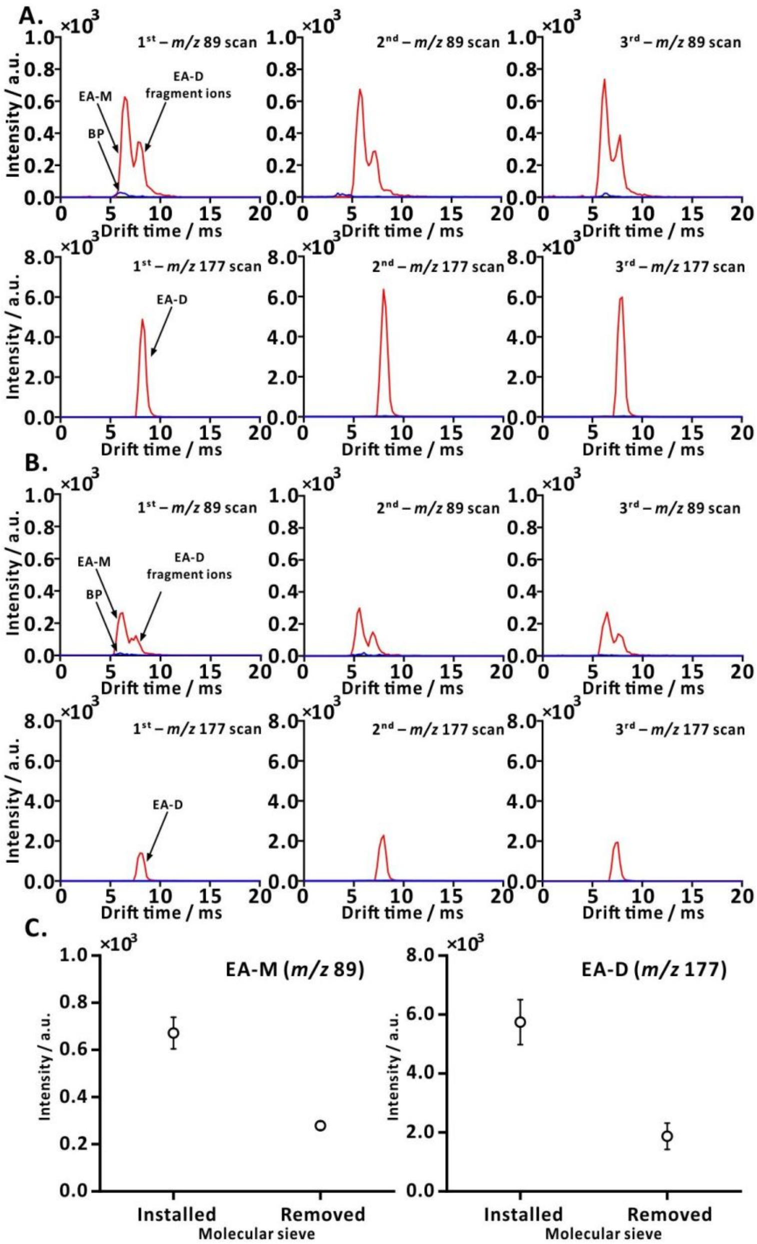

Figure S13. Effect of moisture on results obtained by APCI-COME-IM-QqQ-MS. Ethyl acetate monomer and dimer signal performance with (A) molecular sieve tube installed; (B) molecular sieve tube removed; (C) MS signal intensity comparison of installing and removing molecular sieve tube. BP, blank peak; EA-M, ethyl acetate monomer; EA-D, ethyl acetate dimer. Ethyl acetate monomer (*m/z* 89) and dimer (*m/z* 177) are based on two separate scans with one event (event time, 0.103 s).

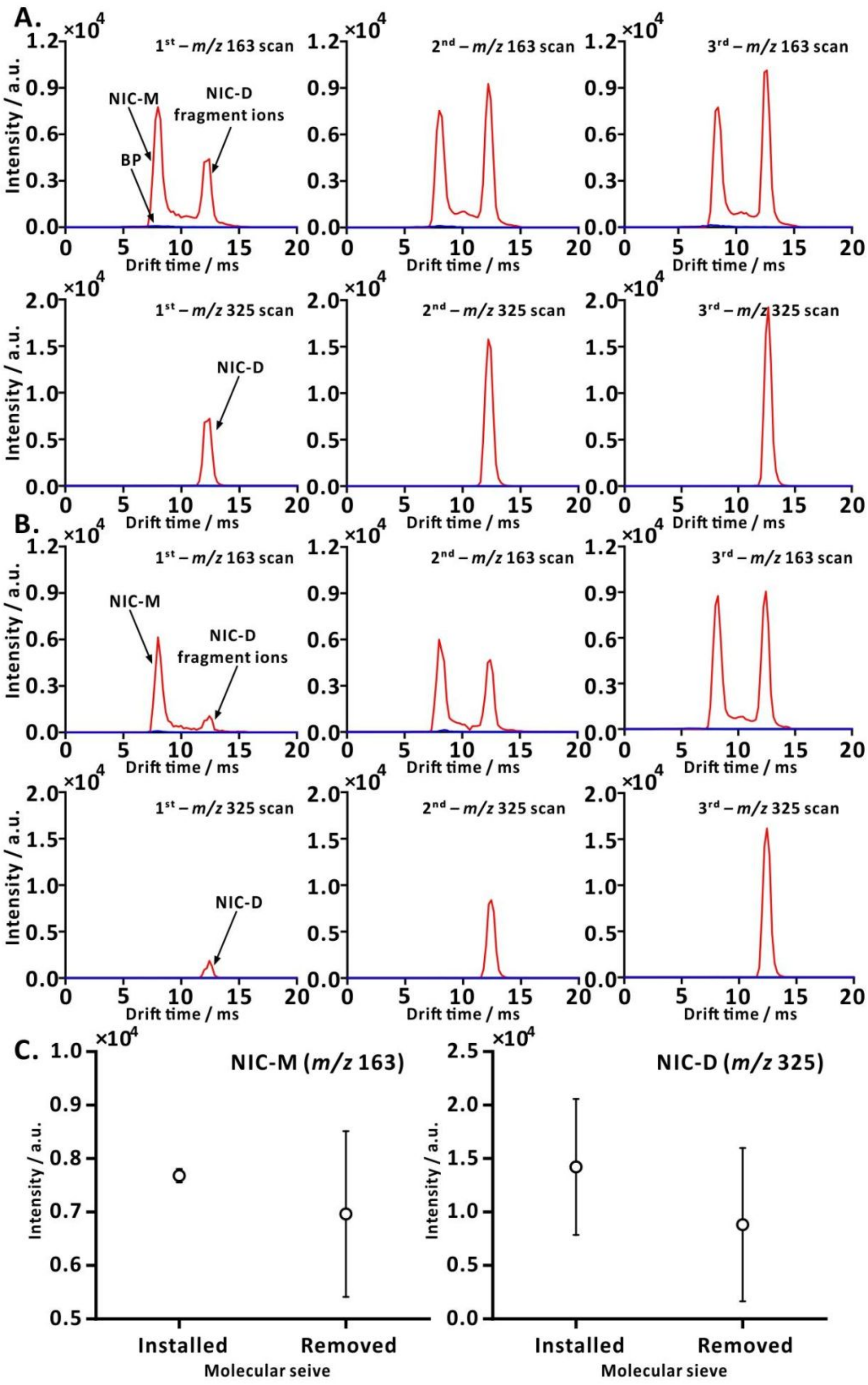

Figure S14. Study of influence of moisture on SESI-COME-IM-QqQ-MS results. Nicotine monomer and dimer signal performance with (A) molecular sieve tube installed and (B) molecular sieve tube removed. (C) MS signal intensity comparison of installing and removing molecular sieve tube. BP, blank peak; NIC-M, nicotine monomer; NIC-D, nicotine dimer. Two events were configured in SIM mode (event time, 0.206 s).

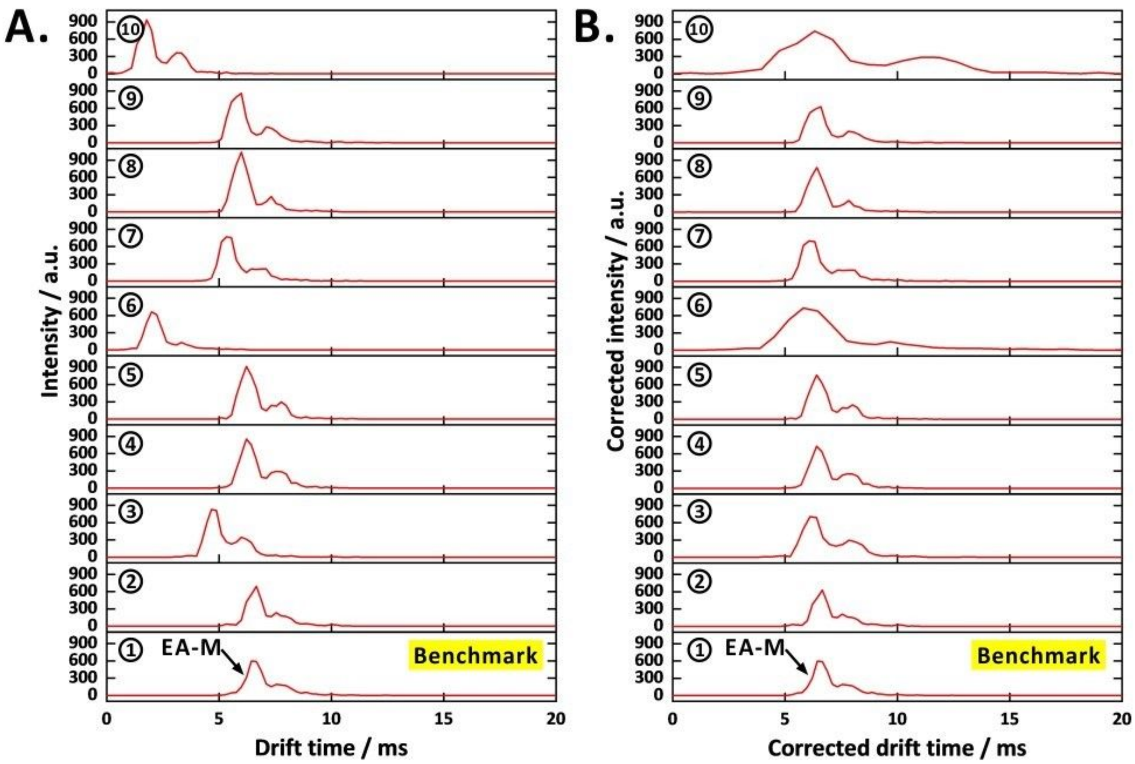

Figure S15. Drift time and intensity correction of SIM-IM spectra (*m/z* 89) of ethyl acetate monomer via isotope internal standard in repeatability test: (A) before correction; (B) after correction. EA-M, ethyl acetate monomer. APCI voltage, 19 kV; 100 µL of 1.5×10^{-2} M ethyl acetate (before dilution) and 100 µL of 1.5×10^{-2} M isotopically labelled ethyl acetate (before dilution) were mixed with 2 mL of water and 100 mg of manganese (IV) oxide. Four events were configured in SIM mode (event time, 0.412 s).
